# Investigating the Association between COMMD3 Expression and the Prognosis of Hepatocellular Carcinoma

**DOI:** 10.7150/jca.62454

**Published:** 2022-03-21

**Authors:** Wei Cheng, Ziwei Cheng, Chunsheng Zhang, Lingling Weng, Dongwei Xing, Minguang Zhang

**Affiliations:** 1 Shanghai Municipal Hospital of Traditional Chinese Medicine, Shanghai University of Traditional Chinese Medicine, Shanghai 200071, China.; 2 First Affiliated Hospital of Anhui Medical University, Hefei, Anhui 230022, China.; 3 Nanjing Hospital of Chinese Medicine affiliated to Nanjing University of Chinese Medicine, Nanjing 210001, China.; 4 Luan Hospital of Chinese Medicine affiliated to Anhui University of Chinese Medicine, Luan 237001, China.

**Keywords:** COMMD3, mRNA, prognosis, hepatocellular carcinoma

## Abstract

The Copper Metabolism MURR1 Domain (COMMD) family proteins are known to play roles in promoting or inhibiting the proliferation, migration and invasion of tumor cells. However, the role of COMMD3 in hepatocellular carcinoma are still unclear. By investigating the TCGA datasets, we found that the mRNA expression of COMMD3 was significantly upregulated in hepatocellular carcinoma tissue compared with normal liver tissue, which was further supported by Oncomine dataset, Western blot, qRT-PCR, and IHC analysis. Moreover, Kaplan-Meier survival analysis showed that the high expression of COMMD3 was associated with poor overall survival (OS) and disease-free survival (DFS). Consistently, the clinic-pathological analysis found that the overexpression of COMMD3 was correlated with advanced TNM stage, advanced T stage and vascular invasion. By performing multivariate analysis, we found that the expression of COMMD3 was an independent influencing factor on OS and DFS. Furthermore, we knocked down COMMD3 in HCC cells via RNA interference. The results showed that silencing COMMD3 could inhibit the migration, invasion, and angiogenesis of HCC cells. Finally, we established xenograft tumor model in nude mice, and the knockdown of COMMD3 suppressed tumor growth and angiogenesis. In summary, our study showed that the high expression of COMMD3 was correlated with poor prognosis in HCC patients and contributed to migration, invasion and angiogenesis of HCC cells.

## Introduction

Hepatocellular carcinoma (HCC) is a common malignant tumor with relatively high morbidity and mortality, which poses a serious threat to human health 1. Early stage HCC patients who undergo surgical treatment can obtain relatively better survival rate, but most HCC patients diagnosed at advanced or locally advanced stage lose the chance of surgical treatment and usually exhibit very poor therapeutic effect 2. Recently, several studies showed that some genes that were upregulated in HCC tissue were associated with poor prognosis and promoted invasion and metastasis of HCC 3-6. Therefore, these genes might serve as potential targets for HCC treatment.

The COMMD (Copper Metabolism MURR1 Domain) Protein Family is highly conserved in eukaryotic multicellular organisms. It consists of 10 families, and is involved in the regulation of several biological processes through protein-protein interaction networks (PPI) 7-9. COMMD1, the most representative family number, was the first discovered COMMD protein 9. COMMD1 is a multifunctional regulatory protein and takes part in copper homeostasis, ionic transport and secretion, oxidative stress and protein aggregation, NF-κB-mediated transcription, hypoxia induced transcription, DNA damage response, and oncogenesis 10-13. COMMD3, COMMD4, and COMMD6 are also thought to interact with NF-κB subunits and downregulate NF-κB-mediated transcription, but their effects are relatively weaker compared with COMMD1 14. COMMD3 is also involved in proliferation, migration and invasion of prostate cancer cells, leading to poor prognosis in patients with metastatic prostate cancer 15. COMMD5, also known as hypertension-related calcium-regulated gene (HCaRG), has been shown to inhibit proliferation of tumor cell 16-18. COMMD7 can also activate CXCL10 expression by regulating NF-κB and reactive oxygen species 19. COMMD9 can inhibit cell proliferation and migration, arrest cell cycle at G1/S transition, and induce autophagy in non-small cell lung cancer cells 20. COMMD10 is a relatively common COMMD protein, which can inhibit invasion and metastasis of colorectal cancer by regulating NF-κB pathway 21.

At presently, there are also some studies published successively, which are involved in values of COMMD family proteins in HCC. COMMD1 was showed to maintain the amount of Atp7b (ATP-dependent copper transporter 7β) and favor recruitment of Atp7b from cytoplasmic vesicles to the trans-Golgi network membrane in a mouse hepatoma cell line 22. Upregulation of COMMD1/4 and downregulation of COMMD2/3/7/8/9 were significantly associated with good OS in HCC patients 23. COMMD7 knockdown or COMMD1 overexpression can inhibit proliferation, migration, and invasion of HCC stem cells (HCSCs) through suppression of NF-kB p65 24. COMMD7 also promotes proliferation, migration and invasion of HCC cells through regulating CXCL10 25-27. COMMD8 was upregulation in HCC tissue and restoration of COMMD8 significantly rescued the proliferation, migration and invasion of HCC cells transfected with sh-MNX1-AS1 28. COMMD10 can suppress proliferation and promote apoptosis of HCC cells through inhibiting NF-κB signaling and value up BCLC staging in predicting OS in HCC patients 29.

Despite the known roles of COMMD family proteins, the correlation between the potential prognostic value of COMMD3 expression level in HCC patients remains largely unknown. The purpose of this study was to explore the expression level and function of COMMD3 in HCC cells and analyze the relationship between COMMD3 expression and HCC prognosis.

## Materials and methods

### Patients

The study included 374 HCC patients with COMMD3 mRNA expression information from The Cancer Genome Atlas (TCGA), of which 366 had complete OS information, and 318 had complete DFS information. Baseline demographic, survival data, clinicopathological characteristics, and genetic data were obtained through the TCGA public website. The primary endpoints were OS and DFS. OS was defined as the time interval between the date of diagnosis and the date of death from any cause or the last follow-up. DFS was defined as the time interval between the date of diagnosis and the date of the first documented evidence of relapse, progression, death, or the last follow-up. All patients signed informed consent, and the study protocol was approved by the Washington University Human Studies Committee. The Roessler Liver 2 datasets were obtained from the Oncomine database (https://www.oncomine.org) 30. The immunohistochemistry (IHC) data of COMMD3 protein in HCC tissues and normal liver tissues were downloaded from The Human Protein Atlas (http://www.proteinatlas.org).

### Sample information from TCGA

According to the inclusion criteria 31: (1) Histologic diagnosis was HCC. (2) There were no any treatment performed for their disease (such as: ablation, chemotherapy, or radiotherapy) before resection of samples. (3) Institutional review boards at each tissue source site reviewed protocols and consent documentation and approved submission of cases to TCGA. (4) All case stages were performed according to the American Joint Committee on Cancer (AJCC). (5) Each frozen primary tumor specimen had a companion normal tissue specimen (blood or blood components, including DNA extracted at the tissue source site). Adjacent tissue was submitted for some cases. (6) Specimens were shipped overnight using a cryoport that maintained an average temperature of less than -180°C. (7) Tumor samples with ≥ 60% tumor nuclei and ≤ 20% or less necrosis were submitted for nucleic acid extraction.

### Materials and reagents

Dulbecco's modified Eagle's medium (DMEM) and penicillin/streptomycin were purchased from Hyclone (Logan, USA). Foetal bovine serum (FBS) was purchased from Gibco (South America). Anti-COMMD3 polyclonal antibodies and anti-CD34 monoclonal antibodies were purchased from Bioss (Boston, USA). Anti-HIF1α monoclonal antibodies and GAPDH monoclonal antibodies were purchased from CST (Boston, USA). Anti-VEGF monoclonal antibodies were purchased from RD (Minneapolis, USA). Horseradish peroxidase (HRP)-conjugated secondary antibodies, New Super ECL Assay kit, and anti-β-actin monoantibodies were purchased from KeyGEN BioTECH (Nanjing, China). Transwell chambers and Matrigel were purchased from Corning Life Sciences (8-μm pores, Tewksbury, MA, USA) and BD Biosciences (San Jose, CA, USA), respectively. The qRT-PCR kit was purchased from Takara (Shiga, Japan). The BCA Protein Assay Kit was purchased from Beyotime Biotechnology (Shanghai, China). The recombinant lentiviral expression vectors (LV-Con and LV-ShCOMMD3), and polybrene were purchased from Genechem (Shanghai, China). BALB/c nude mice were purchased from Shanghai SLAC Experimental Animal Co., Ltd (Shanghai, China).

### Cell culture

Human umbilical vein endothelial cells (HUVECs), normal liver cell (L-02) and two types of HCC cell lines (SK-Hep1 and Hep-3B) were obtained from the Type Culture Collection of the Chinese Academy of Sciences (Shanghai, China), and they were cultured in DMEM containing 10% FBS and 1% penicillin-streptomycin at 37 °C and 5% CO2.

### *In vitro* transfection

LV-ShCOMMD3 (sequence: GACCAATCAACTTCATAGGAT) is lentiviral vector expressing GFP and shRNA against COMMD3. LV-Con (sequence: TTCTCCGAACGTGTCACGT) is control lentiviral vector expressing GFP alone. For lentivirus infection, 2×10^5^ HCC cells/well were plated into six-well culture plates. 24 hours later, 2.6 µl of LV-ShCOMMD3 (8×10^8^ TU/ml) virus or 3 µl of LV-CON (7×10^8^ TU/ml) virus, and 1 µl of polybrene (10 mg/ml) were mixed in 1.0 ml of DMEM containing 10% FBS without antibiotics. The old culture medium was removed, and the mixture containing the lentivirus and polybrene were added into each culture well and gently mixed. After 12 h, the cell culture medium was removed, and 2 ml of DMEM containing 10% FBS and antibiotics were added. After 72 h of incubation, infection was conformed under fluorescence microscopy. The infected HCC cells were cultured continuously or collected immediately for qRT-PCR assay or western blotting.

### qRT-PCR Assay

Total RNA of the cultured cells was extracted using TRIzol Reagent according to the manufacturer's instruction. The qRT-PCR kit and 0.5µg total RNA were used for qRT-PCR assays. All the primers were synthesized by Sangon Biotech. The primer sequences are listed in Table 1. These experiments were repeated three times.

### Western blotting analysis

The cells were lysed with RIPA lysis buffer containing protease inhibitors and phenylmethylsulfonyl fluoride for 30 min on ice. The protein concentration was determined using a BCA protein assay kit. Equal amounts of proteins (50-100 μg) were loaded onto SDS-PAGE and then transferred onto a nitrocellulose membrane. Subsequently, the membranes were blocked with 5% non-fat milk at room temperature for 2 h and then incubated with primary antibodies (1:1,000) overnight at 4 °C, followed by incubation with HRP-conjugated secondary antibodies (1:2,000) at room temperature for 1 h. Finally, immunoreactive bands were visualized using a super ECL kit.

### Wound healing assay

Cells were plated in six-well plates. When cells were at 80-90% confluence, a straight trace was scratched in the middle of each well with a white sterile pipette tip on the cell layer. Then, the cells were rinsed three time with fresh serum-free DMEM to wash away cell debris. These cells were cultured for 24 h and imaged with an inverted microscope at 0 h and 24h. This experiment was repeated three times.

### Transwell invasion assay

The bottom chambers of the transwell apparatus were filled with 600 µL of DMEM supplemented with 20% FBS. The top chambers of transwell apparatus were coated with 100 µL of diluted matrigel (1: 3) for 30 min at 37 °C, and then 5×10^4^ HCC cells were plated. The plates were incubated at 37 °C for 72 h. The invaded cells were analysed by crystal violet staining. Images were acquired using inverted microscope, and invaded cells were quantified by manual counting. This experiment was repeated three times.

### HUVECs tube formation assay

The medium of human HCC cells infected with lentiviral constructs (LV- ShCOMMD3 and LV-Con) were collected as conditioned medium for subsequent analysis. Each well of the 96-well plates was coated with 50 µL matrigel and incubated at 37 °C for 30 min. Then, 100 µL of conditioned medium was added. HUVECs (2.5×10^4^ cells) were seeded on the matrigel and incubated at 37 °C. After 24 h of incubation, the tubular structure formation in HUVECs was imaged, and the tube number was quantified using Image J. This experiment was repeated three times.

### *In vivo* xenograft model assay

In order to validate the function of COMMD3, we carried out the *in vivo* xenograft model assay. Eight male BALB/c nude mice (four-six weeks old) were randomly divided into two groups (LV-Con and LV-ShCOMMD3 groups). After one week of adaptive feeding, the two groups were subcutaneously inoculated with 5×10^6^ transformed Sk-Hep1 cells (transfected with LV-Con-Sk-Hep1 or LV-ShCOMMD3-Sk-Hep1) in a volume of 100 µl on the right flank. The dimension of the xenograft tumours was measured every seven days for five consecutive weeks, and the size was calculated using the formula V = 0.5×L×W^2^ (V, volume; L, length; W, width). All animals were sacrificed by euthanasia and the xenograft tumours were harvested for the IHC assay. All animal experiments involved in our study were performed according to guidelines of institutional animal care and use committee (IACUS) and approved by the Ethics Committees of Shanghai Municipal Hospital of Traditional Chinese Medicine, Shanghai University of Traditional Chinese Medicine.

### IHC staining

Paraffin-embedded sections were deparaffinized in an oven, hydrated with ethanol, and incubated with 3% H_2_O_2_ to block endogenous peroxidase activity, followed by antigen retrieval. After cooling, the sections were blocked with 10% normal goat serum for 1 h and then incubated with primary antibodies against COMMD3, HIF-1α, VEGF and CD34 (diluted at 1: 200) in 10% normal goat serum for 1 h. Next, the sections were incubated with HRP-conjugated secondary antibodies (1:600) for 1 h. Finally, all sections were counterstained with diaminobenzidine and Mayer's haematoxylin. The evaluation of IHC staining was mainly based on intensity (0: no intensity; 1: weak intensity; 2: moderate intensity; 3: strong intensity) and percentage (0: less than 10%; 1: between 10% - 25%; 2: between 25% - 50%; 3: more than 50%) of positive cells. According to the total score (staining intensity plus positive cell score), the results were divided into the negative expression group (total score: 0-2) and the positive expression group (total score: 3-6).

### Statistical analysis

SPSS software 24.0 and GraphPad Prism software 7.0 were used to carry out all statistical analyses. Descriptive statistics was used to describe clinicopathological characteristics of HCC patients. Data sets were described with median and/or range. The Mann-Whitney U test and chi-square test were used to compare numerical data and categorical data, respectively. The Kaplan-Meier methods and log-rank test were used to estimate survival analysis. Univariate and multivariate Cox proportional hazard models were established for OS and DFS, using a limited backward elimination process. Experiment data were presented as mean ± standard deviation, and the significance of comparison was estimated by the Student's t test. *P* < 0.05 was considered as statistically significant.

## Results

### COMMD3 expression level in HCC tissue and normal liver tissue

**Figure 1:**
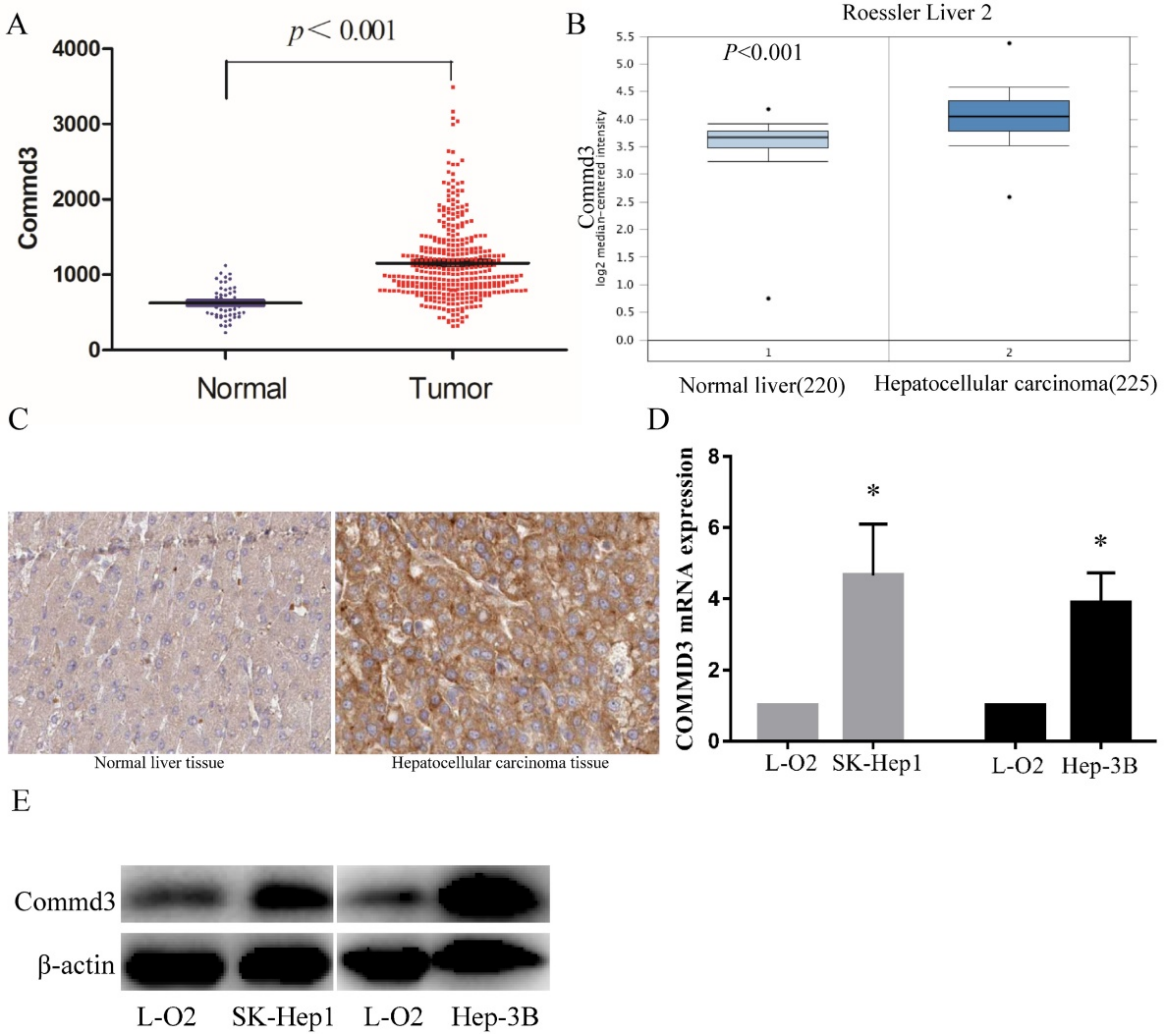
** The COMMD3 expression in HCC tissue vs normal liver tissue, or HCC cells lines vs normal liver cell line. (A)** COMMD3 mRNA levels in HCC tissue and normal liver tissue from TCGA. **(B)** COMMD3 mRNA levels in HCC tissue and normal liver tissue from Oncomine. **(C)** COMMD3 protein levels in HCC tissue and normal liver tissue from The Human Protein Atlas. **(D)** COMMD3 mRNA levels from HCC cells lines and normal liver cell line. **(E)** COMMD3 protein levels from HCC cells lines and normal liver cell line.

To further explore the clinical significance of COMMD3 in HCC, we compared COMMD3 mRNA levels in HCC tissue and normal liver tissue using TCGA dataset and Oncomine dataset. Our results showed that the mRNA levels of COMMD3 in HCC tissue were significantly higher than that in normal liver tissue (Figure 1A, B), which was further supported by IHC analysis from The Human Protein Atlas (Figure 1C). Moreover, we found that the mRNA and protein levels of COMMD3 in HCC cells (SK-Hep1 and Hep-3B) were higher than that in normal liver cells (L-02) (Figure 1D, E).

### Clinical-pathological characteristics of HCC patients

The clinical-pathological characteristics of all HCC patients from TCGA dataset are listed in Table 2. 366 HCC patients with complete OS information and 318 HCC patients with complete DFS information were included in our study. Among the patients, 169 were under 60 years old, 252 were male, and 182 were Caucasian. The number of patients in G1, G2, G3, and G4 stage were 55, 178, 124, and 12, respectively. The number of patients with TNM stage I, stage II, stage III and stage IV was 173, 88, 86, and 6, respectively. The number of patients with T stage T1, T2, T3 and T4 was 182, 95, 81, and 13, respectively. The number of patients with macrovascular invasion, microvascular invasion and no vascular invasion was 16, 94, and 208, respectively.

### The effect of COMMD3 expression on OS and DFS in HCC patients

**Figure 2:**
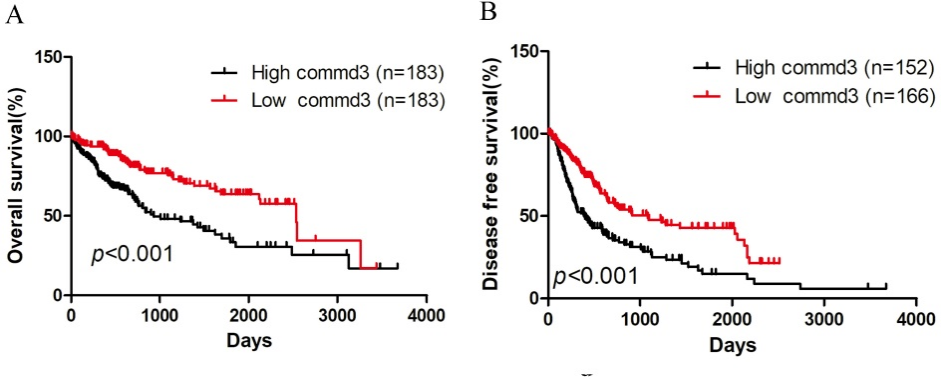
** Kaplan-Meier curves of HCC patients grouped by COMMD3 mRNA levels. (A)** High expression level of COMMD3 mRNA was associated with poor OS in HCC patients. **(B)** High expression level of COMMD3 mRNA was associated with poor DFS in HCC patients.

To evaluate the effect of COMMD3 expression level on OS and DFS in HCC patients, we performed Kaplan-Meier survival analysis. All HCC patients with OS information were divided into high COMMD3 expression group and low COMMD3 expression group based on the median expression level of COMMD3 mRNA. Patients with COMMD3 mRNA level ≥ median were classified as high expression group, and the others were included in the low expression group. The results showed that high COMMD3 mRNA expression was associated with poor OS (Figure 2A) and DFS (Figure 2B).

### The correlation between clinical-pathological characteristics of HCC patients and COMMDs expression level

To investigate the effect of COMMD3 expression on the clinical-pathological characteristics of HCC patients, we analyzed the relationship between clinical-pathological characteristics and COMMD3 mRNA level. The results showed that high levels of COMMD3 mRNA in HCC patients was linked with advanced TNM stage, advanced T stage, and vascular invasion (Table 3).

### Multivariate analyses of OS and DFS in HCC patients

To further estimate the prognostic value of COMMD3 mRNA level for HCC patients, we performed multivariate analysis on COMMD3 mRNA level (high vs. low), age (≥60 vs. <60 years), sex (man vs. female), race (white vs. others), G stage (G3+G4 vs. G1+G2), TNM stage (III+ IV vs. I+ II), T stage (T3+T4 vs. T1+T2), and vascular invasion (Yes vs. No) (Table 4 and Table 5). The results showed that COMMD3 mRNA level was one of the independent risk factors for OS and DFS.

### The effect of COMMD3 inhibition on cell migration, invasion and angiogenic ability of HCC cells

**Figure 3:**
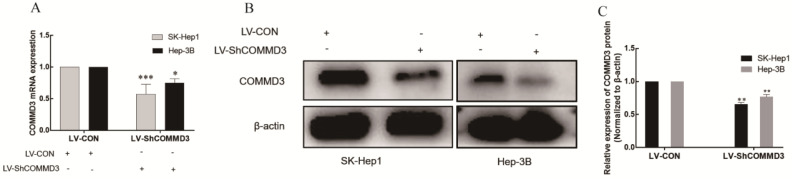
** Establishment of the stably transformed SK-Hep1 and Hep-3B cell lines with COMMD3 shRNA. (A)** COMMD3 mRNA levels in SK-Hep1 and Hep-3B cell lines transfected with COMMD3 shRNA. (B) The levels of COMMD3 proteins in SK-Hep1 and Hep-3B cell lines transfected with COMMD3 shRNA. **(C)** The relative expression levels of COMMD3 proteins in the SK-Hep1 and Hep-3B cell lines transfected with COMMD3 shRNA were quantified using ImageJ software. Data were plotted with GraphPad Prism 7.0 software. *P<0.05, **P<0.01 and ***P<0.001 compared to the control group.

**Figure 4:**
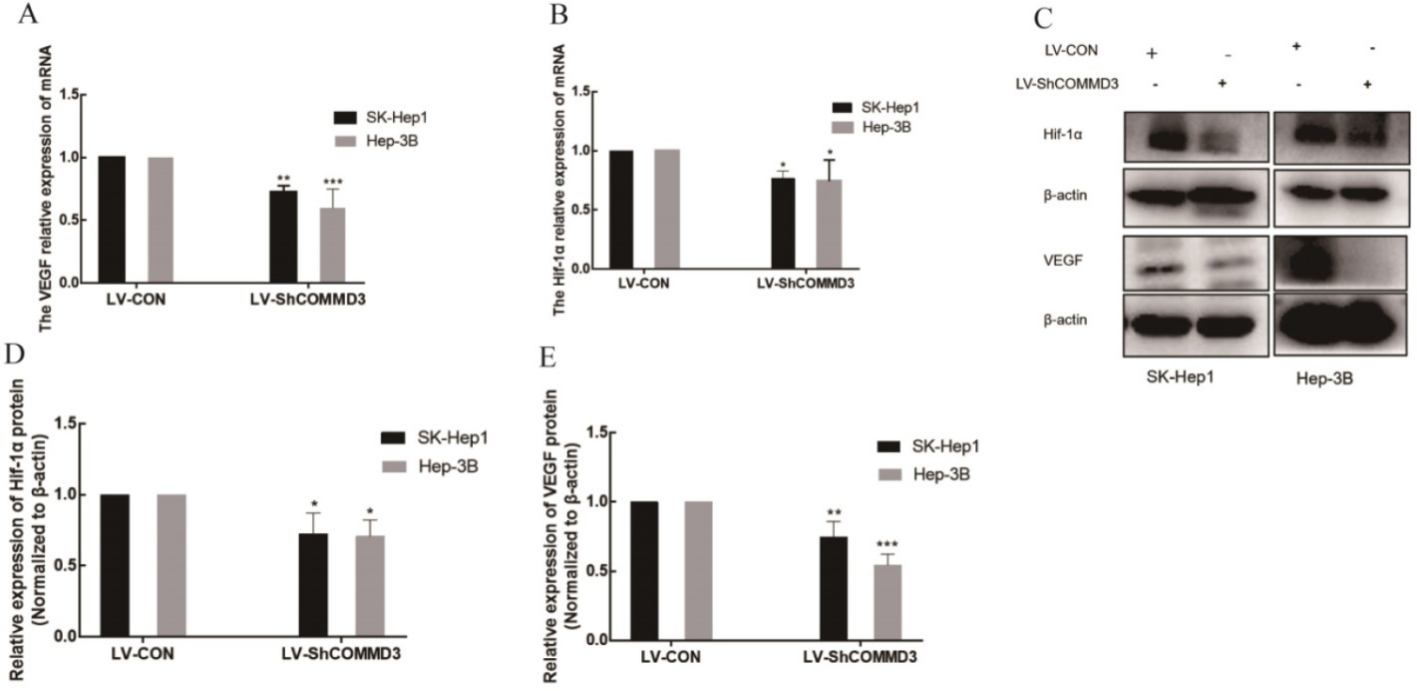
** The effects of COMMD3 knockdown on the mRNA and protein levels of HIF1α and VEGF in HCC cells. (A)** The effects of COMMD3 knockdown on VEGF mRNA levels in SK-Hep1 and Hep-3B cell lines. **(B)** The effects of the COMMD3 knockdown on HIF1α mRNA levels in SK-Hep1 and Hep-3B cell lines. **(C)** The effects of COMMD3 knockdown on protein levels of HIF1α and VEGF protein in SK-Hep1 and Hep-3B cell lines. **(D, E)** The relative expression levels of HIF1α and VEGF proteins in SK-Hep1 and Hep-3B cell lines with COMMD3 shRNA were quantified using ImageJ software. Data were plotted using GraphPad Prism 7.0 software. *P<0.05, **P<0.01 and ***P<0.001compared to the control group.

To investigate the effects of COMMD3 on migration, invasion and angiogenic ability of HCC cells, we transformed SK-Hep1 and Hep-3B HCC cells lines with constructs expressing COMMD3 shRNA (LV-SHCOMMD3) or control constructs (LV-Con). We confirmed that the mRNA and protein levels of COMMD3 in LV-SHCOMMD3 transformed cells were significantly down-regulated compared to LV-Con group (Figure 3A, B, and C). Additionally, we also found that the mRNA and protein levels of VEGF and HIF1α were downregulated in LV-ShCOMMD3 group (Figure 4A-E). Thus, we successfully made stable SK-Hep1 and Hep-3B cell lines in which COMMD3 expression was inhibited via RNA interference. To examine the effects of COMMD3 inhibition on the migration and invasion abilities of SK-Hep1 and Hep-3B cells, we performed wound healing assay and transwell invasion assay. The results showed that COMMD3 knockdown significantly suppressed the migration (Figure 4A, B) and invasion (Figure 4C, D) abilities of SK-Hep1 and Hep-3B cells. Moreover, we found that the conditioned medium from the LV-ShCOMMD3 group could significantly inhibit the angiogenesis of HUVECs, as compared with the medium from LV-Con group (Figure 4E, F).

### RNA interference-mediated COMMD3 silencing suppressed the growth of subcutaneous xenograft tumours in nude mice

**Figure 5:**
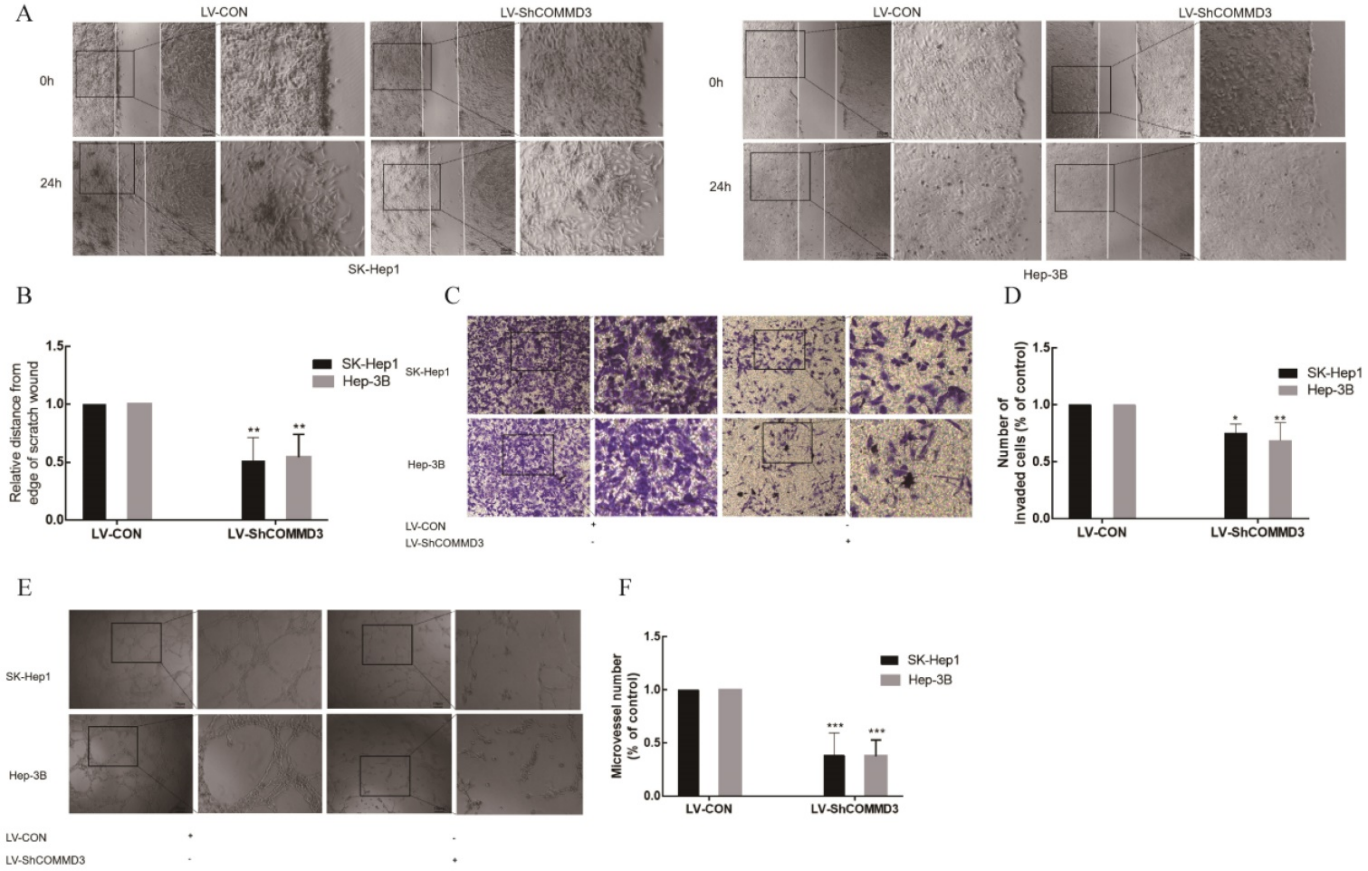
The inhibitory effects of COMMD3 knockdown on the migration, invasion and angiogenic ability of HCC cells. **(A)** The wound healing assay used for testing cell migration ability; Scale bar, 20 µm in A. **(B)** The histogram represents the migration distance relative to the control at 24 h after scratching the cell layer. **(C)** The transwell assay used for testing cell invasion. Scale bar, 10μm in C.** (D)** The histogram represents the average number of invasive cells relative to the control. **(E)** The tube formation assay used for testing HCC cell-induced angiogenesis in HUVECs. Scale bar, 10μm in E.** (F)** The area covered by the tube network was quantified using the Image-Pro Plus software. Data were plotted with the GraphPad Prism 7.0 software. *P<0.05, **P<0.01 and ***P<0.001compared to the control group. 2.5 times magnification in the right side of all original figures.

In order to further illustrate the function of COMMD3, we established xenograft tumor model using BALB/c nude mice. The stably transformed Sk-Hep1 cells (LV-Con-Sk-Hep1 or LV-ShCOMMD3-Sk-Hep1) were injected subcutaneously onto the right flank of the nude mice. The size of the xenograft tumours was measured every seven days for five weeks. The results showed that the xenograft tumours from mice injected with LV-Con cells were significantly larger than those injected with LV-ShCOMMD3 cells (Figure 5A, B). The IHC and western blotting results showed that expression levels of COMMD3, HIF1α, VEGF and CD34 proteins in xenograft tumours from LV-Con group were significantly higher than those from LV-ShCOMMD3 group (Figure 5C, D).

## Discussion

HCC is a common malignant tumor with relatively high morbidity and mortality, more than half of which were first diagnosed and treated in China 32. Although many strategies have been developed to treat HCC, their efficacies are still unsatisfactory 33. Sorafenib, a first-line treatment drug for advanced HCC, can suppress growth of liver tumor by inhibiting Raf-1, B-Raf, VEGFRs 1, 2, and 3 and PDGFR-β 34, 35. However, the median OS of sorafenib for advanced HCC is only 10.7 months 36. Therefore, it is in urgent need to explore new targets for the treatment of HCC.

COMMDs family consist of 10 numbers, most of which have the function of regulating NF-κB. Some members of COMMDs family are associated with tumor progression. COMMD1 can disrupt HIF-1α/β dimerization and inhibit human tumor cell invasion 11. COMMD1 also promotes inflammation and protects mice from colitis and colitis-associated cancer 37. COMMD5 promotes renal cell migration via a TGF-α autocrine loop mechanism 38. COMMD5 also inhibits proliferation of renal carcinoma cell and gastric cancer cell 16-18. COMMD7 can inhibit cell growth, migration and invasion of HCC cells through PI3K-AKT and ERK/MAPK signal pathways 39, 40. COMMD7 also inhibits migration and invasion of HCC stem cells via regulating mesenchymal-epithelial transition 41. COMMD9 can inhibit cell proliferation and migration, and induce autophagy in non-small cell lung cancer cells 20. COMMD10 can suppress invasion and metastasis of colorectal cancer by regulating NF-κB pathway 21. However, the effects of COMMD3 on HCC cells are still unclear. Thus, it is necessary to investigate the effects of COMMD3 on the survival of HCC patients and the migration, invasion and angiogenesis of HCC cells.

Our study found that the mRNA and protein levels of COMMD3 in HCC tissue and HCC cells were significantly upregulated. Kaplan-Meier survival analysis showed that high level of COMMD3 mRNA was associated with poor OS and DFS in HCC patients. Moreover, high COMMD3 mRNA level was associated with advanced TNM stage, advanced T stage, and vascular invasion in HCC patients. Multivariate analysis showed that high COMMD3 level was an independent risk factor for OS and DFS, while advanced T stage was only an independent risk factor for DFS in HCC patients. Silencing COMMD3 by RNA interference could significantly suppress migration, invasion and angiogenesis of HCC cells. Finally, the xenograft tumour assay using nude mice showed that COMMD3 knockdown inhibited tumor growth and the expression of HIF1α, VEGF and CD34. Our study also suggested that COMMD3 might an upstream regulator of HIFα in HCC cells.

In this study, we showed, for the first time, that COMMD3 was an independent risk factor for OS and DFS in HCC patients. The results suggested that COMMD3 might be a potential target for treating HCC. We speculated that the poor prognosis of HCC patients with high COMMD3 expression might be due to the promoting effect of COMMD3 on cell migration, invasion and angiogenesis. In the future study, we need to further determine the function of COMMD3 through *in vitro* and *in vivo* assays, as well as to find drugs that can inhibit the expression of COMMD3.

Our study still has some limitations. Firstly, the survival analysis was only based on TCGA database, and more databases are needed, such as GEO. Secondly, we still need a lot of clinical data to further verify the results. Lastly, the results need to be confirmed in other HCC cell line tests, such as Huh7, bel7404, and SMMC-7721 cell lines.

## Conclusions

Our study showed that COMMD3 was upregulated in HCC tissues and HCC cells, and its overexpression was associated with poor OS and DFS in HCC patients. We also showed that COMMD3 could promote the migration, invasion, and angiogenic ability of HCC cells.

## Figures and Tables

**Figure 6:**
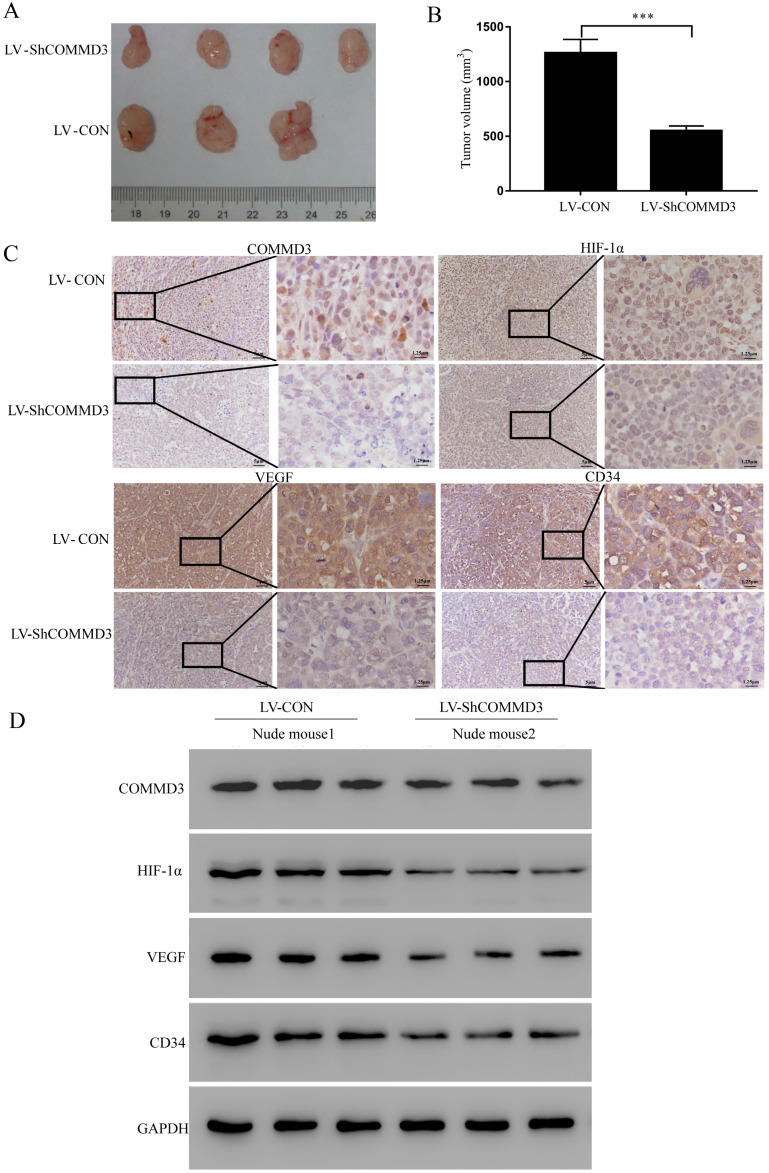
RNA interference-mediated COMMD3 knockdown suppressed the growth of subcutaneous xenograft tumours in nude mice.** (A)** Images of subcutaneous xenograft tumours harvested from the nude mice. **(B)** Comparison of the size of xenograft tumours from mice at five weeks after they were inoculated with SK-Hep1 cells with LV-CON or LV- ShCOMMD3. Data were expressed as the means ± SDs.** (C)** IHC staining for the COMMD3, HIF1α, VEGF and CD34 proteins in the xenograft tumours (×200 and ×800).** (D)** Western blotting analysis on COMMD3, HIF1α, VEGF and CD34 proteins in the xenograft tumours from mice injected with cells from the LV-CON and LV-ShCOMMD3 groups. Data were plotted with the GraphPad Prism 7.0 software. ***, *P*<0.001 compared to the control group.

**Table 1:** Primers used for QRT-PCR

Name	Sequences (5'-3')
GAPDH-F	CAGGAGGCATTGCTGATGAT
GAPDH-R	GAAGGCTGGGGCTCATTT
COMMD3-F	ACTCCAACGCCTTCACGCTTC
COMMD3-R	GGATGATCTAACACGGCCTCGTC
HIF-1α-F	TGACAAGCCACCTGAGGAGA
HIF-1α-R	ACACGCGGAGAAGAGAAGGA
VEGF-F	TACCTCCACCATGCCAAGTG
VEGF-R	GGTCTCGATTGGATGGCAGT

**Table 2:** Clinicopathologic characteristics of HCC patients

Characteristics	Median (range) or N/%
Age/years, median (range)	61(17-90)
**Age group/n (%)**	
< 60 years	169 (45.3%)
≥ 60 years	204(54.7%)
**Gender/n (%)**	
Male	252 (67.4%)
Female	122 (32.6%)
**Race/n (%)**	
White	182 (50.0%)
Asian	161 (44.2%)
Black	19 (5.2%)
American Indian	2 (0.6%)
**G stage/n (%)**	
G1	55 (14.9%)
G2	178 (48.2%)
G3	124 (33.6%)
G4	12 (3.3%)
**TNM stage/n (%)**	
I	173 (49.0%)
II	88 (24.9%)
III	86 (24.4%)
IV	6 (1.7%)
**T stage/n (%)**	
T1	182 (49.1%)
T2	95 (25.6%)
T3	81 (21.8%)
T4	13 (3.5%)
**Vascular invasion/n (%)**	
Macrovascular invasion	16 (5.0%)
Microvascular invasion	94 (29.6%)
None	208 (65.4%)

HCC, hepatocellular carcinoma. The case numbers of clinicopathologic characteristics are not available, which including 1 for age, 10 for race, 5 for G stage, 21 for TNM stage, 3 for T stage, and 56 for vascular invasion.

**Table 3:** Correlation between COMMD3 expression level and clinical-pathological variables in HCC patients

Characteristics	High commd3	Low commd3	χ2	*P* value
**Age group/n (%)**			0.79	0.374
< 60 years	80 (43.0%)	89 (47.6%)		
≥ 60 years	106 (57.0%)	98 (52.4%)		
**Gender/n (%)**			1.216	0.270
Male	131 (70.1%)	121 (67.4%)		
Female	56 (29.9%)	66 (32.6%)		
**Race/n (%)**			1.099	0.294
White	84 (47.2%)	98 (52.7%)		
Others	94 (52.8%)	88 (47.3%)		
**G stage/n (%)**			0.154	0.695
G1+ G2	118 (64.1%)	115 (62.2%)		
G3+ G4	66 (35.9%)	70 (37.8%)		
**TNM stage/n (%)**			5.728	0.017
I+ II	121 (68.4%)	140 (79.5%)		
III+ IV	56 (31.6%)	36 (20.5%)		
**T stage/n (%)**			4.747	0.029
T1+T2	129 (69.7%)	148 (79.6)		
T3+T4	56 (30.3.9%)	38 (20.4%)		
**Vascular invasion/n (%)**			10.111	0.001
Yes	65 (43.6%)	45 (26.6%)		
No	84 (56.4%)	124 (73.4%)		

HCC, hepatocellular carcinoma.

**Table 4:** Cox proportional-hazard regression analysis for OS in HCC patients

Variables	Univariable analysis		Multivariable analysis	
	HR (95%CI)	*P*-value	HR (95%CI)	*P*-value
COMMD3 (high vs. low)	2.292 (1.592-3.302)	≤0.001	1.772 (1.170-2.683)	0.007
Age (≥60 vs. <60 years)	1.213 (0.848-1.734)	0.291		
Sex (man vs. female)	1.230 (0.856-1.770)	0.263		
Race (white vs. others)	1.255 (0.869-1.814)	0.226		
G stage (G3+G4 vs. G1+G2)	1.076 (0.743-1.558)	0.698		
TNM stage (III+ IV vs. I+ II)	2.485 (1.703-3.625)	≤0.001	0.976 (0.129-6.990)	0.903
T stage (T3+T4 vs. T1+T2)	2.592 (1.812-3.709)	≤0.001	2.362 (0.321-17.380)	0.399
Vascular invasion (Yes vs. No)	1.345 (0.879-2.059)	0.172		

OS, Overall survival; HCC, hepatocellular carcinoma; HR, hazard ratio; CI, confidence interval.

**Table 5:** Cox proportional-hazard regression analysis for DFS in HCC patients

Variables	Univariable analysis		Multivariable analysis	
	HR (95%CI)	*P*-value	HR (95%CI)	*P*-value
COMMD3 (high vs. low)	2.011 (1.490-2.714)	≤0.001	1.782 (1.344-2.426)	≤0.001
Age (≥60 vs. <60 years)	1.035 (0.772-1.388)	0.818		
Sex (man vs. female)	1.178 (0.864-1.607)	0.301		
Race (white vs. others)	1.314 (0.993-1.740)	0.056		
G stage (G3+G4 vs. G1+G2)	1.022 (0.764-1.366)	0.886		
TNM stage (III+ IV vs. I+ II)	1.726 (1.275-2.336)	≤0.001	1.004 (0.660-1.444)	0.982
T stage (T3+T4 vs. T1+T2)	2.240 (1.633-3.072)	≤0.001	2.023 (1.271-3.218)	0.003
Vascular invasion (Yes vs. No)	2.009 (1.425-2.833)	≤0.001	1.066 (0.847-1.340)	0.588

DFS, disease free survival; HCC, hepatocellular carcinoma; HR, hazard ratio; CI, confidence interval.
